# A Review of Conductive Carbon Materials for 3D Printing: Materials, Technologies, Properties, and Applications

**DOI:** 10.3390/ma14143911

**Published:** 2021-07-13

**Authors:** Yanling Zheng, Xu Huang, Jialiang Chen, Kechen Wu, Jianlei Wang, Xu Zhang

**Affiliations:** 1 Fujian Science & Technology Innovation Laboratory for Optoelectronic Information of China, Fuzhou 350108, China; zhengyanling@fjirsm.ac.cn; 2 College of Chemistry and Materials Science, Fujian Normal University, Fuzhou 350007, China; 3 CAS Key Laboratory of Design and Assembly of Functional Nanostructures, Fujian Key Laboratory of Nanomaterials, Fujian Institute of Research on the Structure of Matter, Chinese Academy of Sciences, Fuzhou 350002, China; 4 Fujian College, University of Chinese Academy of Sciences, Fuzhou 350002, China; 5 Fujian Universities and Colleges Engineering Research Center of Modern Facility Agriculture, Fujian Polytechnic Normal University, Fuzhou 350300, China; 6 School of Mechanical & Automotive Engineering, Fujian University of Technology, Fuzhou 350118, China; huangxu@fjut.edu.cn; 7 National Garment and Accessories Quality Supervision Testing Center (Fujian), Fujian Provincial Key Laboratory of Textiles Inspection Technology, Fujian Fiber Inspection Center, Fuzhou 350026, China; cjliang@yeah.net; 8 Fujian Key Laboratory of Functional Marine Sensing Materials, Minjiang University, Fuzhou 350108, China; wkc@fjirsm.ac.cn; 9 Innovation Center for Textile Science and Technology, State Key Laboratory for Modification of Chemical Fibers and Polymer Materials, College of Materials Science and Engineering, Donghua University, Shanghai 201620, China

**Keywords:** 3D printing, conductive carbon materials, polymer composites, functional devices

## Abstract

Carbon material is widely used and has good electrical and thermal conductivity. It is often used as a filler to endow insulating polymer with electrical and thermal conductivity. Three-dimensional printing technology is an advance in modeling and manufacturing technology. From the forming principle, it offers a new production principle of layered manufacturing and layer by layer stacking formation, which fundamentally simplifies the production process and makes large-scale personalized production possible. Conductive carbon materials combined with 3D printing technology have a variety of potential applications, such as multi-shape sensors, wearable devices, supercapacitors, and so on. In this review, carbon black, carbon nanotubes, carbon fiber, graphene, and other common conductive carbon materials are briefly introduced. The working principle, advantages and disadvantages of common 3D printing technology are reviewed. The research situation of 3D printable conductive carbon materials in recent years is further summarized, and the performance characteristics and application prospects of these conductive carbon materials are also discussed. Finally, the potential applications of 3D printable conductive carbon materials are concluded, and the future development direction of 3D printable conductive carbon materials has also been prospected.

## 1. Introduction

Three-dimensional (3D) printing technology is a new manufacturing technology, also known as additive manufacturing (AM), rapid prototyping (RP), and solid freeform fabrication (SFF) [1,2,3], which takes layered manufacturing as the forming principle and integrates advanced technologies such as numerical control, materials science, and computer-aided design. The 3D printing can design and produce objects with complex geometric shapes, which integrates the advanced technologies of computer aided design (CAD), computer aided manufacturing (CAM), computer numerical control machine tools (CNC), laser, precision servo drive, and new materials. According to the 3D design model built on the computer, slicing is carried out to obtain the two-dimensional profile information of each section. The forming head of the 3D printer is in the control system according to the input profile information under the control of the system. The layer of molding material is selectively solidified or cut to form each interface profile, and the stacking between the profiles finally forms a three-dimensional workpiece. Since its birth in the late 1980s, the rapid development of 3D printing technology has made large-scale personalized production possible. The core of the 3D printing process is to transform the 3D model of the complex workpiece to be formed into a simple 2D section combination by slicing. In the process of processing and production, there is no need to use the traditional processing machine tools and models, which simplifies the mold and processing flow needed in the production process, which is very beneficial for scientific research [4] and business [5]. Meanwhile, it is a major innovation for the traditional manufacturing industry [6,7].

With the rapid development of the computer and artificial intelligence industry, the demand for materials with ideal electrical properties is gradually growing. The common conductive materials on sale are inorganic conductive materials and polymer conductive materials, which usually need to be processed before use. The traditional manufacturing methods can be divided into two types according to the forming process: one is the material elimination method, such as cutting, in which the material is constantly reduced in the forming process; the other is the material transfer method, in which the pressure and other methods are used to form the parts in the processing process without changing the quality of the material. Using the traditional processing method, we need to use a variety of machines, go through multiple steps, and even need to produce the corresponding mold to obtain the final products [8]. The 3D printing technology has many advantages compared with the process of traditional conductive products, which could develop new products quickly without using various auxiliary tools in a very short time. In the face of the rapid changes in the market and the shortening of the product life cycle, it can shorten the trial production time of new products, which can not only reduce the production cost, rapid prototyping, and improve the manufacturing speed, but also improve the production efficiency high-response speed to market of enterprises giving them opportunities in fierce market competition.

Conductive materials are the most important type of 3D printing functional materials, which have many potential applications such as electrodes [9,10,11], wearable devices [12,13], sensors [12,14], etc. Conductive materials for 3D printing mainly include metal, carbon, and polymer composites. By doping different conductive carbon materials in the different polymer matrices, the composites have conductive properties. Using different types of 3D printing technology, the required conductive products can be prepared quickly. Although metal filler has good conductivity, it is expensive and oxidizes easily losing its conductivity, which limits the application of metal filler in 3D printable conductive materials. Compared with metal materials, the carbon filler has high stability and good conductivity, which is easier to process and has also been widely concerned [15]. Many reports and reviews have been concerned with 3D printable conductive carbon materials. However, there is still a lack of review on the relationship between 3D printing methods and conductive carbon materials. Moreover, there are still some deficiencies in the induction and collation of various new types of 3D printable conductive carbon materials in recent years. For this reason, this review firstly systematically introduces the common conductive carbon materials and 3D printing materials. Then, the conductive carbon materials based on 3D printing technology and their applications are emphasized, hoping to provide some useful references for further research on the 3D printable conductive carbon materials in the future.

## 2. Common Conductive Carbon Materials

Carbon materials are more common and widely used, which are closely related to human life. With the increasing attention to carbon materials, the research on carbon materials is more and more in-depth. Different carbon materials can be formed according to the electronic orbital arrangement and crystallization modes of carbon elements, such as carbon black (CB), carbon fiber (CF), carbon nanotubes (CNTs), graphene (GE or GP), graphite sheet (GNS), and other carbon materials [16]. However, carbon materials can also be divided into zero dimension (0D, CB), one-dimension (1D, including CNTs, CF), two-dimension (2D, GE or GP), and three-dimension (3D, GNS) [17,18,19,20]. Because of the regular arrangement of carbon atoms, carbon materials have many advantages, such as high conductivity, chemical stability, low density, excellent mechanical properties, and so on. Due to the excellent electrical conductivity, carbon materials are often used as conductive fillers and composites with a polymer matrix to make insulating polymer materials conductive. In addition, they can also give the composites good processability and mechanical properties.

### 2.1. Carbon Black

Carbon black (CB) is the most common carbon material which is lightweight, and has a low price and good conductivity. It is the first carbon material used as conductive filler and polymer composite, and it is also the most widely used conductive filler in industry [21,22]. The research results show that the smaller the size, the more complex the structure, and the less the surface-active groups of the carbon black particles, the better the conductivity of the composites. The traditional filling method requires a large amount of carbon black to make the composite from insulation to conduction. However, the high filling amount makes the processing of the composite more difficult and the mechanical properties of the composite lower, which is bad for production and processing [23]. At present, the research on CB is not the increase of traditional dosage, but to improve the electrical conductivity of composites by reducing the threshold quality of CB and changing the filling method.

**Figure 1:**
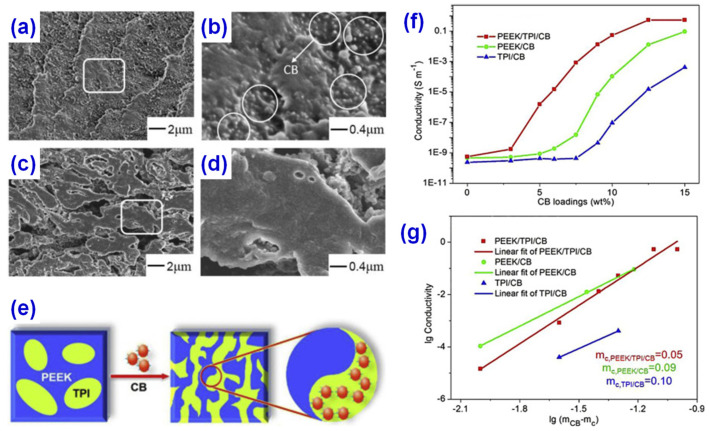
Scanning electron microscopy (SEM) of (**a**) un-etched fractured surfaces of poly (ether ether ketone/polyimide/carbon black (PEEK/TPI/CB)-5wt%, (**b**) enlarged SEM of the white marked region of (**a**), (**c**) etched fractured surface of PEEK/TPI/CB-5wt%, and (**d**) magnified SEM of the white marked region of (**c**). (**e**) Scheme of CB induced co-continuity of PEEK/TPI blends. (**f**) Effect of CB content on the conductivity of the composites at 10^3^ Hz. (**g**) Linear fit of conductivity of the composites. Reprinted with permission from Ref. [24]. Copyright 2015 Elsevier and Copyright Clearance Center.

The percolation threshold of composites can be reduced by constructing a double percolation structure, which is a common method to construct conductive polymer composites. In the classical double permeable structure, the conductive fillers are selectively located in one phase of the blend, and only the permeable conductive network is formed in the insulating polymer material. For example, Gao et al. [24] used CB, poly (ether ether ketone) (PEEK), and polyimide (TPI) as raw materials to construct a double permeable structure. The properties of conductive polymer composites were adjusted by controlling the position of CB in the PEEK/TPI matrix through melt blending. The percolation threshold decreased from about 10 wt% to 5 wt%, as shown in Figure 1.

**Figure 2:**
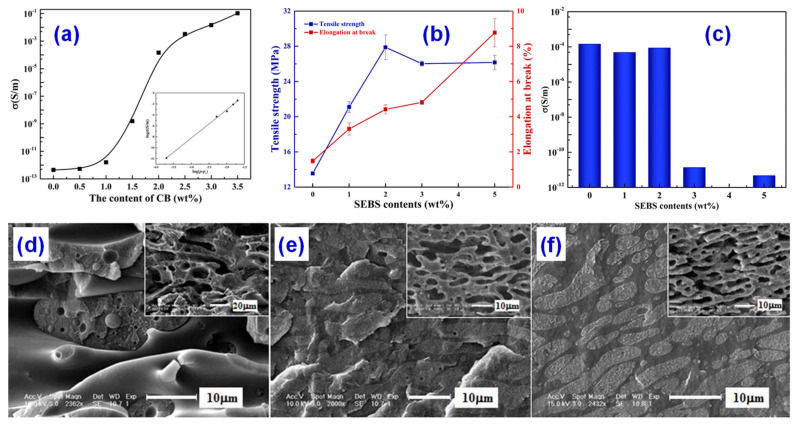
(**a**) Electrical conductivity of CB/polystyrene (PS)/polypropylene (PP) composites as a function of CB content. The more CB content, the higher conductivity. Effect of styrene-ethylene/butane-styrene (SEBS) content on the (**b**) tensile properties (strength and elongation at break) and (**c**) electrical conductivity of CB/SEBS/PS/PP composites. SEM micrographs of CB/SEBS/PS/PP with different SEBS contents: (**d**) 0 wt%, (**e**) 1 wt%, and (**f**) 2 wt%. Reprinted with permission from Ref. [25] Copyright 2017 Elsevier and Copyright Clearance Center. The ratio of PS/PP keeps 60/40.

However, when conducting networks are constructed in immiscible polymer blends, the interfacial interaction between immiscible phases is poor and the mechanical properties become worse. To balance the mechanical and electrical properties of immiscible polymer blends, Chen [25] used a double percolation structure, where CB was added when the mass ratio of polystyrene (PS)/polypropylene (PP) was 60/40, and the percolation conductive structure was formed in a specific phase. The perfect double percolation structure was constructed, which greatly reduced the percolation threshold. The mechanical properties of the composites were modified by adding the fourth component styrene-ethylene/butane-styrene (SEBS). When the content of SEBS reaches 2 wt%, the conductive polymer composites keep a double permeability structure and have good electrical properties. At the same time, the SEBS can also make the conductive polymer composites have good mechanical properties. Finally, the electrical and mechanical properties of the composites are well balanced, as presented in Figure 2.

### 2.2. Carbon Fiber

**Figure 3:**
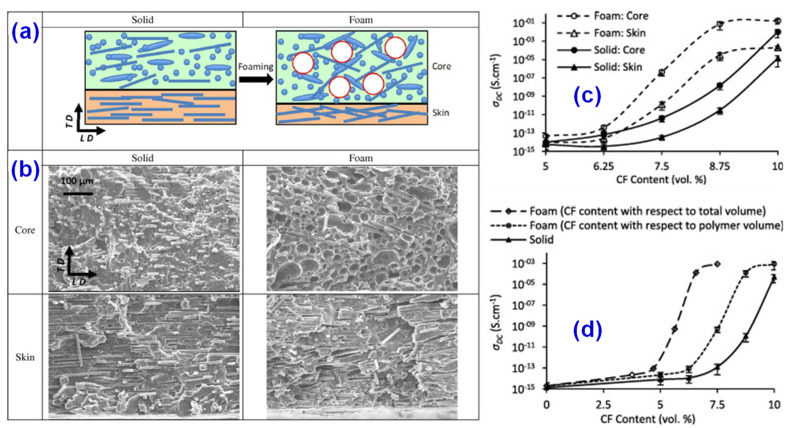
(**a**) Effects of foaming on the structure of injection-molded composites, where LD and TD represent the length and thickness directions, respectively. (**b**) SEM images of solid’s core, foam’s core, solid’s skin, and foam’s skin for PP/carbon fiber (CF) composites with 10 vol% of CF. The foamed sample has a large void. (**c**,**d**) Effect of foaming and CF content on the through-plane DC electrical conductivity of PP/CF composites, the more content of CF, the higher conductivity. Reprinted with permission from Ref. [28] Copyright 2013 Elsevier and Copyright Clearance Center.

Carbon fiber (CF) is a special fiber mainly composed of carbon elements [26]. The molecular structure of CF is between graphite and diamond. Since the Japanese scientist Shindo [27] made carbon fiber from polyacrylonitrile (PAN) in 1959, CF has already developed into an independent and complete industrial system. In addition to lightweight, good fiber degree, and strong tensile strength, CF also possesses the characteristics of high electrical and thermal conductivity of common carbon materials. Thanks to the variety of advantages, carbon fiber materials are widely used in modern industry. For instance, CF is often used as a conductive filler to prepare conductive polymer composites, and the morphology and surface area of the CF has a great influence on the conductive properties of the composites. The properties of polypropylene/carbon fiber (PP/CF) composite foams with different loading of CF were studied by Ameli [28] and co-workers. As shown in Figure 3, the density of the composites decreases by about 25%, while the volume fraction of the percolation threshold decreases from 8.5% to 7%. Furthermore, the dielectric constant increases, which further improves the electrical properties of PP/CF composites.

### 2.3. Carbon Nanotubes

**Figure 4:**
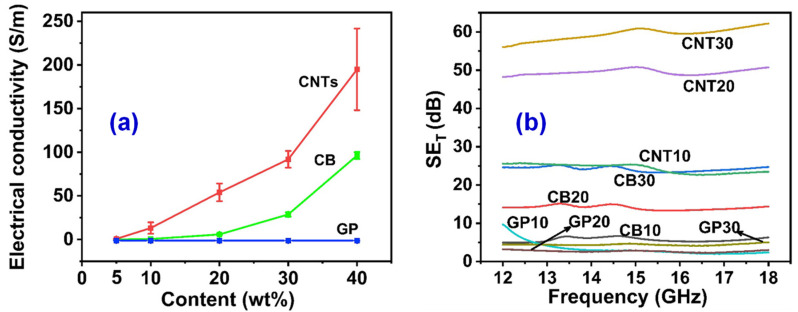
(**a**) Effect of filler content on the electrical conductivity of isoprene rubber (IR)-based composites filled with different carbon fillers (0d CB, 1D carbon nanotubes (CNTs), and 2D graphene (GP)). (**b**) EMI shielding effectiveness of IR-based composites filled with different dimensional carbon fillers. Compared with other carbon materials, CNT has the best shielding effect, and the shielding effect is the best when the content of CNT is 30%. Reprinted with permission from Ref. [41] Copyright 2020 Elsevier and Copyright Clearance Center.

In 1991, Japanese electron microscope scientist Iijima first discovered carbon nanotubes (CNTs) in the process of preparing C60 by an arc method [29]. Their unique tubular structure can be seen as a hollow cylinder rolled up by graphite sheets, which is a one-dimensional hollow conductive carbon material with a large aspect ratio. The larger aspect ratio and specific surface area make CNTs disperse unevenly in the matrix, which agglomerates easily and causes stress concentration. The special structure endows CNTs with excellent mechanical [30,31,32], electrical [33,34,35], and chemical properties [36,37], which has been widely used in nano-electronic devices, field emission, composite reinforced materials, and so on [38,39]. Cheney et al. [40] found that the introduction of ionic CNTs into epoxy matrix can improve the uneven distribution of CNTs. When the loading rate of CNTs reaches 8 wt%, the shear viscosity decreases by 69.1%. Wang et al. [41] studied the effect of carbon filler dimension (0d CB, 1D CNTs, and 2D GP, etc.) on the properties of flexible electromagnetic interference (EMI) shielding materials based on isoprene rubber (IR) matrix composites. Due to the large-aspect-ratio and outstanding electrical conductivity, the 1D CNTs are easier to form the perfect conductive network inside IR matrix, exhibiting that the IR/CNTs composites have superior electrical conductivity than those of the IR/CB and IR/GP composites at the same loading, as presented in Figure 4a. It can be concluded from Figure 4b that the EMI shielding properties depend on their electrical properties: the higher the electrical conductivity, the higher the EMI shielding effectiveness value.

### 2.4. Graphene

**Figure 5:**
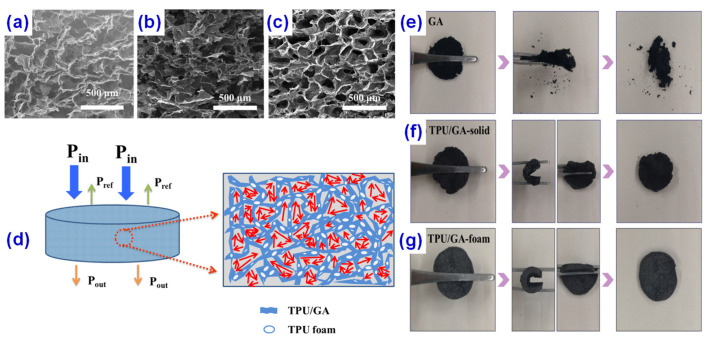
SEM of (**a**) graphene aerogel (GA), (**b**) thermoplastic polyurethane (TPU)/GA-solid, and (**c**) TPU/GA-foam. (**d**) Sketch of the microwave transfers across the TPU/GA composite foams. Optical images of flexibility of (**e**) GA, (**f**) TPU/GA-solid, and (**g**) TPU/GA-foam. The toughness of the sample with graphene is greatly enhanced after foaming treatment, and the resilience of the sample is also enhanced. Reprinted with permission from Ref. [51] Copyright 2020 Elsevier and Copyright Clearance Center.

Graphene [42,43] (GE or GP) is the thinnest two-dimensional material found at present, which was first obtained by the mechanical stripping method by the Geim group [44] in 2004. Graphene is the basic structure of other graphite materials with excellent physical and chemical properties. The strength of graphene is the highest among all tested materials, which is 100 times that of steel [45], and its carrier mobility is over 10 times that of commercial silicon wafer [46]. In addition, graphene also has room temperature quantum Hall effect, room temperature ferromagnetism, and other special properties [47]. These excellent properties have triggered a new round of research. The graphene aerogels (GAs) with excellent electrical conductivity and large specific surface area are ideal materials for conductive fillers. Li et al. [48] have prepared the anisotropic graphene aerogels (AGAs) with highly aligned graphene networks by a directional-freezing followed by a freeze-drying process, which exhibited different microstructures and performances along the axial (freezing direction) and radial (perpendicular to the axial direction) directions. It was found that the epoxy-based composites with 0.8 wt% thermally annealed GAs have an EMI shielding effectiveness of 27 dB, and the epoxy-based composites with 0.8 wt% thermally treated anisotropic AGAs have an enhanced EMI shielding effectiveness of 32 dB along the radial direction with a slightly decreased shielding effectiveness of 25 dB along the axial direction. However, the direct introduction of conductive fillers into the polymer matrix makes it easy to increase the density of the polymer/GA composites, which is not conducive to the later preparation. Polymer foaming has been validated as an effective way to fabricate lightweight functional polymer composites [49,50]. Jiang et al. [51] prepared thermoplastic polyurethane/graphene aerogel (TPU/GA) composites with super-low density by supercritical CO_2_ foaming, where the porous structures were introduced into the composites, as shown in Figure 5. When the GA content is only 2 wt%, the conductivity of the composites reaches up to 50 S/m and the EMI shielding value is about 34.3 dB, resulting from the interconnected graphene networks in TPU/GA composite foams. In addition, the cellular or porous structure provides more absorption paths and improves the electromagnetic shielding coefficient.

## 3. 3D Printing of Conductive Carbon Materials

Recently, the types of 3D printers are emerging endlessly [52]. There are nearly 20 different process systems in the field of 3D printing at home and abroad. Among them, six processes are the most widely used and the most mature technology. They are fused deposition modeling (FDM), selective laser sintering (SLS), selective laser melting (SLM), selective laser melting (SLM), stereolithography apparatuses (SLA), laminated object manufacturing (LOM) and three-dimensional printing (3DP). The principles and materials used in these processes are different. However, the suiTable 3D printing method for conductive carbon materials mainly includes FDM [53,54], SLS [55,56], SLA [57], and so on. The choice of 3D printing method depends on the application fields and the processing restrictions of the products, such as the concentration conditions of printing materials, and the properties of loaded fillers, etc. The selection of 3D printing parameters and the design and manufacture of the printing structure will also have a certain impact on the performance of the final products [58,59,60]. Usually, the 3D printing method is selected according to the product characteristics and the raw material properties. The following contents of this review would focus on the conductive carbon materials for 3D printing, which are classified into four subsections according to the 3D printing method, including FDM, SLS, digital light processing (DLP), and other printing technologies.

### 3.1. Fused Deposition Modelling

**Figure 6:**
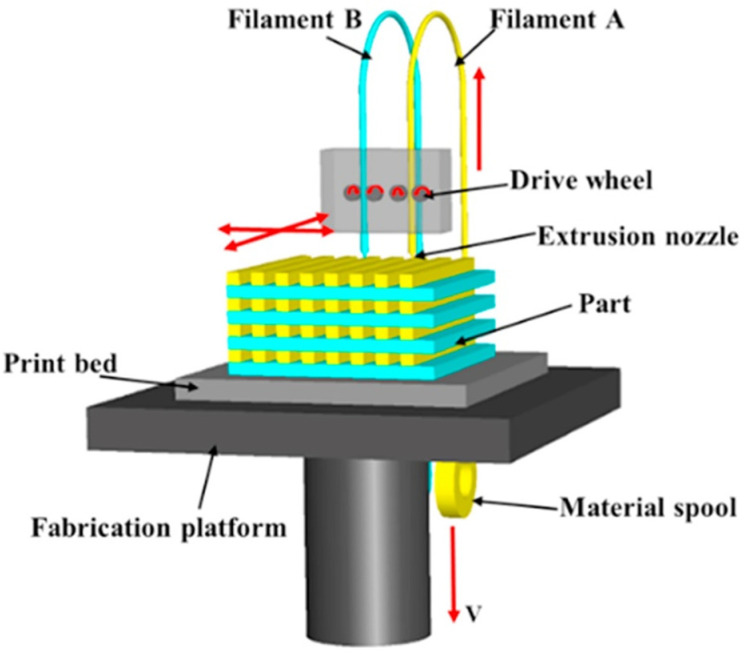
Schematic diagram of dual nozzle fused deposition modeling (FDM) equipment. Reprinted with permission from Ref. [65] Copyright 2017 Elsevier and Copyright Clearance Center.

In 1988, Scott Crump invented FDM and founded STRATASYS. In addition, printer manufacturers such as Ultimaker, Markforge, XYZprinting, Zortax, German RepRap and Dagoma are constantly improving their printers. Nowadays, FDM is one of the most common and widely used 3D printing technologies. The principle of FDM is to control the temperature of the nozzle through the computer, while the thermoplastic material filament is pushed by the driver wheel. After melting at the nozzle, the material melt is extruded and deposited on the panel or the material that has been former cooled and solidified. Under the control of the computer, the final product is formed by stacking layer by layer [60,61]. Usually, the FDM process needs to make supporting structures at the same time in the process of making production. To lower material cost and improve production efficiency, newly developed FDM equipment adopts double nozzles for production, as illustrated in Figure 6. After the material passes through the twin-screw extruder, filament-shaped (round filamentous, 1.75 mm in diameter) thermoplastic polymer is formed through the winding after extrusion. The program software is prepared in advance and the required relevant parameters are set, and the required samples can be printed out through the FDM 3D printer. The FDM 3D printing technology is easy to operate, low cost, and has high material utilization, which is one of the most commonly used 3D printing technologies [62]. The disadvantage of the FDM 3D printing technology is that there are obvious stripes on the surface of the finished product, and the connection between layers is weak [63,64,65]. The conductive composites prepared with conductive carbon filler have excellent mechanical and conductive properties and have great application prospects in structural electronics, supercapacitors, and multi-shape conductive electrodes, etc. At the same time, based on the advantages of FDM, such as fast forming speed, the high utilization rate of raw materials, and forming any complex parts, we can use FDM to produce personalized customized products to meet the different needs of individuals for different products. Using the traditional RepRap FDM 3D printer, Christopher et al. [66] fabricated and printed the electrochemical energy storage architecture of PLA/graphene filament, to explore its possibility as a potential graphene-based lithium-ion anode and solid-state graphene supercapacitor, providing a simple and cheap alternative for traditional lithium-ion batteries. In addition, by testing the electrochemical hydrogen production capabilities of these devices, the filament is expected to replace the platinum-based electrode (an electrolytic cell). Christopher [66] proposed that the 3D printing of conductive filament based on graphene allows the simple manufacturing of energy storage devices through customization and conceptual design, which lays the foundation for the extended application of the material. In addition, the industrial 3D printer produced by the Markforge company adopts fused filament fabrication (FFF). Its principle is the same as that of FDM. It can print and add carbon fiber and other reinforced material wires, which directly enhances the performance of printing products [67,68,69].

**Figure 7:**
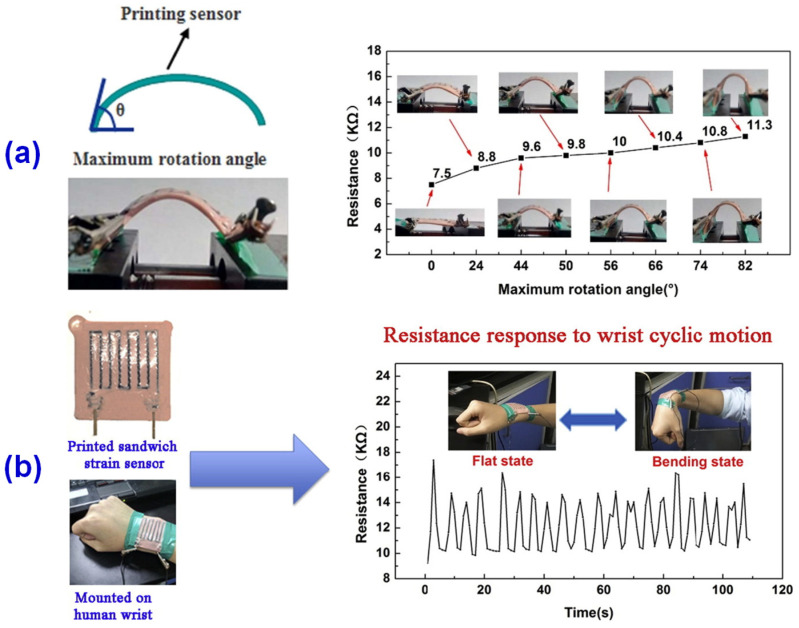
Resistance of sandwich strain sensor (**a**) corresponding to different bending angles and (**b**) response to wrist cyclic motion between flat and bending state. Reprinted with permission from Ref. [70] Copyright 2018 Elsevier and Copyright Clearance Center.

In terms of conductive flexible materials, Huang et al. [70] explored a carbon fiber-filled composite based on the research of conductive rubber by Daver [71] and Lukić [72], and studied its properties. The conductive silicon rubbers (CSR) were prepared by adding 10 phr or 60 phr carbon fiber and a certain amount of thixotropic agent into the base rubber, respectively. The prepared conductive rubber composites were vacuumed to ensure that there was no gap in the 3D printed product. The 3D printed geometry was designed by software in advance, and the product was printed by a desktop FDM 3D printer and further cured by the oven to ensure the stability of electrical and mechanical properties. It was found that the CSR with 5% thixotropic agent has good shape retention [70]. The orientation of carbon fiber in the printing direction endows the product anisotropic electrical and mechanical behavior, where the volume resistivity in the orientation direction (47.3 ± 4.7 Ω cm) was about 6.8 times higher than that in the vertical direction (7.0 ± 0.6 Ω cm). Along the orientation direction, the tensile strength, elongation at break, and Young’s modulus were also greatly improved. The composites show good reversibility under the action of tension, compression, bending, torsion, and cyclic folding. In addition, the sandwich strain sensor was prepared by 3D printing, while the resistance changes with the bending angle (Figure 7a). The installation test results on the human wrist show that the maximum resistance is about 15 kΩ at the bending limit and the initial resistance is about 9 kΩ at the flat wrist state, as shown in Figure 7b.

**Figure 8:**
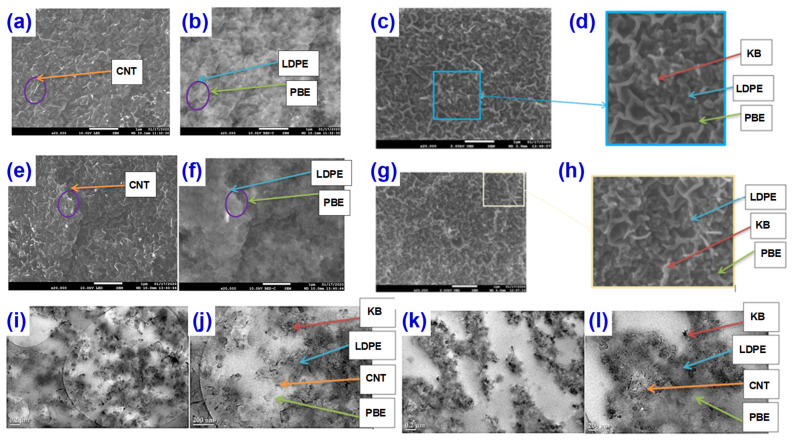
SEM of (**a**–**d**) one- and (**e**–**h**) two-step method. Transmission electron microscopy (TEM) of (**i**,**j**) one- and (**k**,**l**) two-step method. Reprinted from Ref. [83].

In the field of functional textiles, the FDM can realize the integration of conductive tracks and sensors on the surface of textiles, which has attracted much attention [73,74,75,76,77,78,79,80,81,82]. The application of FDM 3D printing technology in functional textiles was explored by Eutionnat-Diffo et al. [83] The immiscible polymer blends were prepared by mixing carbon nanotubes (CNTs) and high structure carbon black (KB) with filled low-density polyethylene (LDPE)/propylene-based elastomer (PBE) in a two-step extrusion process, where the ratio of LDPE/PBE is 60/40 without changing. The SEM and TEM characterizations confirmed that the CNT particles are visualized in both the LDPE and PBE phases and the KB particles in LDPE for one-step extruding process (Figure 8a–d,i,j), while the CNT and KB particles seem to be located at the interface and/or in the LDPE and not in the soft segments of PBE under the two-step extrusion process (Figure 8e–h,k,l). These selective sites play a key role in expanding the common continuity of LDPE and PBE phases in a larger composition range. The enhancement of common continuity in a large range means more stable and accurate information transmission. In addition, the two-step extrusion processing of LDPE/PBE blends filled with carbon particles presented great properties, which could be a better material for functional textile development through 3D printing onto textiles. This shows that the material combined with FDM 3D printing technology can be used to print intelligent clothing with the intelligent response and more comprehensive and systematic detection of human body index. However, more efforts should be made to develop these functional textiles with higher conductivity, flexibility, and mechanical properties.

### 3.2. Selective Laser Sintering

**Figure 9:**
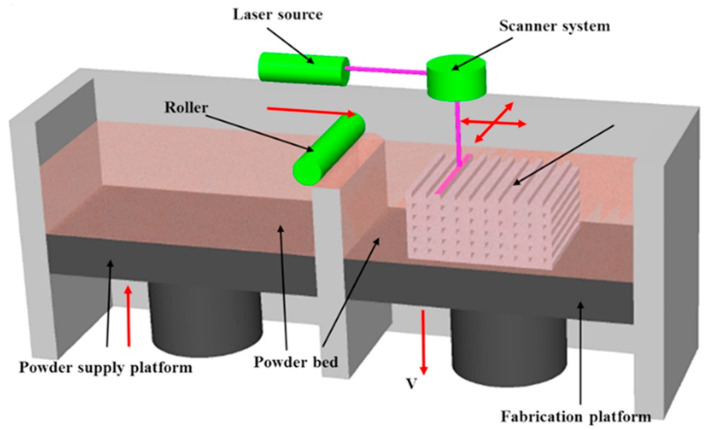
Schematic representation of a typical selective laser sintering (SLS) setup. Reprinted with permission from Ref. [65] Copyright 2017 Elsevier and Copyright Clearance Center.

The SLS process was developed by Dechard in 1989, and then gradually commercialized. This process is commonly used in printing composite materials such as ceramic, glass, fiber, and so on. The principle of the SLS process is that under the control of the computer, the powder supply platform is moved up, and a layer of the powder material is laid on the working platform under the movement of the powder-laying roller. The laser is used to sinter in the selected specific area, and the powder material is melted and bonded by the laser. The fabrication platform descends a little bit, repeating the above steps, and finally forms a printed product. The schematic diagram of SLS 3D printing is shown in Figure 9. The advantage of this process is that there is no need for compaction in the printing process. Compared with conventional injection molding or melt extrusion, SLS does not have such high shear mixing and shear fluidity. These characteristics of SLS limit the ordering of molecular structure, but it can provide a unique way to construct some special structures, such as the separation network structure of fillers. The main disadvantage of the SLS process is the limitation of material use, especially the commercially available SLS printing materials [84]. The future research of the SLS process will be the development of new SLS suitable powder materials and development in the multi-sphere application direction [85].

Although the polymer/nano-filler prepared by traditional solution and melt blending can obtain the composites with uniform dispersion and high mechanical strength. However, it is difficult to form a continuous conductive circuit between the fillers, resulting in low conductivity [86,87,88,89]. The SLS 3D printing process produces the products without shearing and free flow, which means that the SLS can be used to build three-dimensional interconnected conductive structures. Koo et al. [90,91] prepared the polyamide 11 (PA11) composites based on SLS by mixing PA11 with carbon nanotubes and graphene respectively by twin-screw extrusion. The test results show that the flame retardant, thermal properties, and mechanical properties of PA11 are significantly improved with the addition of carbon nanotubes, while the PA11/graphene composites have better thermal stability and conductivity than PA11/CNTs composite. The use of mechanical mixing can also obtain the high conductive performance of SLS composite materials. Athreya et al. [92] prepared the composites by mixing polyamide 12 (PA12) with carbon black through ball milling. The test results show that the best conductivity (10^−2^ S/m) of the PA12/CB composites appears when the carbon black content is 4 wt%. However, the bending strength of PA12 is reduced with the high addition of carbon black.

**Figure 10:**
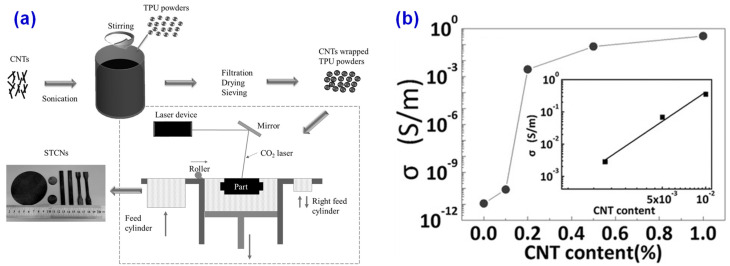
(**a**) SLS 3D printing process of TPU/CNTs composites. (**b**) Electrical conductivity of the SLS 3D printed TPU/CNTs composites as a function of CNTs content, where the inset shows the log-log plot of electrical conductivity versus CNTs content. Reprinted with permission from Ref. [93] Copyright 2017 John Wiley and Sons and Copyright Clearance Center.

The current research on SLS 3D printing is mostly based on polyamide hard materials, but less on flexible materials [60]. To explore the application of flexible materials in SLS 3D printing technology, Li et al. [93] used self-made thermoplastic polyurethane (TPU) materials coated with CNTs to prepare flexible TPU conductor powder for SLS and built a 3D network of conductive isolation by using SLS 3D printing technology. The preparation process is shown in Figure 10a. It was found that the electrical conductivity of TPU/CNTs composites treated with SLS can reach 10^−1^ S/m when the content of CNTs is 1 wt% as presented in Figure 10b, which is seven orders of magnitude higher than that of conventionally injected TPU/CNT composites. The resistance of SLS treated TPU/CNTs composites increased by 40% after 1000 tensile cycles, showing good flexibility and conductivity. The as-prepared composites exhibit potential applications in the fields of 3D printing for flexible circuits, wearable devices, implantable devices, electronic skin, and dielectric elastomer drivers, etc.

### 3.3. Digital Light Processing

**Figure 11:**
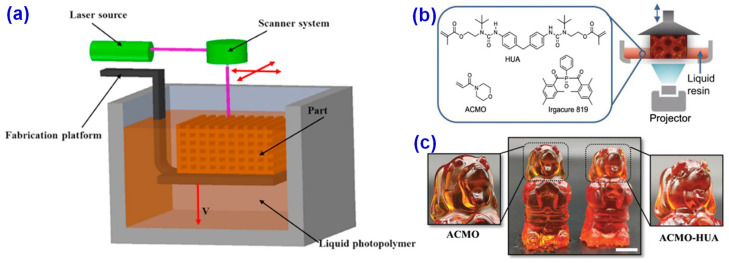
(**a**) The principle of traditional stereolithography apparatus (SLA) printing technology. Reprinted with permission from Ref. [65] Copyright 2017 Elsevier and Copyright Clearance Center. (**b**) The principle of digital light processing (DLP) 3D printing and (**c**) the cartoon dog printed by DLP 3D printing. Reprinted with permission from Ref. [97] Copyright 2020 John Wiley and Sons and Copyright Clearance Center.

DLP is a kind of 3D printing technology based on UV curing. It projects the cross-sectional image of the object into the photosensitive resin to crosslink and solidify, and then solidifies and accumulates layer by layer to form the final product. The core technology of DLP 3D printing is the optical semiconductor and digital microscope device DLP chip invented by Larry Hornback in 1977 [94]. The chip can project a complete image on the screen in coordination with the image signal and light source. Also, the chip can react with high speed and express the fine information of the image well, which enables DLP 3D printing to print high-precision objects [95,96,97]. The material used in DLP 3D printing is the same as that of SLA, but the difference is that SLA uses direct laser beam printing, while DLP 3D printing uses projector to form the surface of resin. SLA printers were first produced in 1988, while DLP 3D printing appeared decades later than SLA. Compared with a traditional SLA (Figure 11a), the DLP 3D printing technology has better image accuracy, less air contact and better product quality, as exampled in Figure 11b,c. In recent years, DLP 3D printing has been widely used in UV curing based 3D printing technology. However, the photosensitive resin used for DLP 3D printing needs low viscosity and low curing shrinkage [95], so the development of photosensitive resin with excellent performance has become a research hotspot of DLP 3D printing technology.

**Figure 12:**
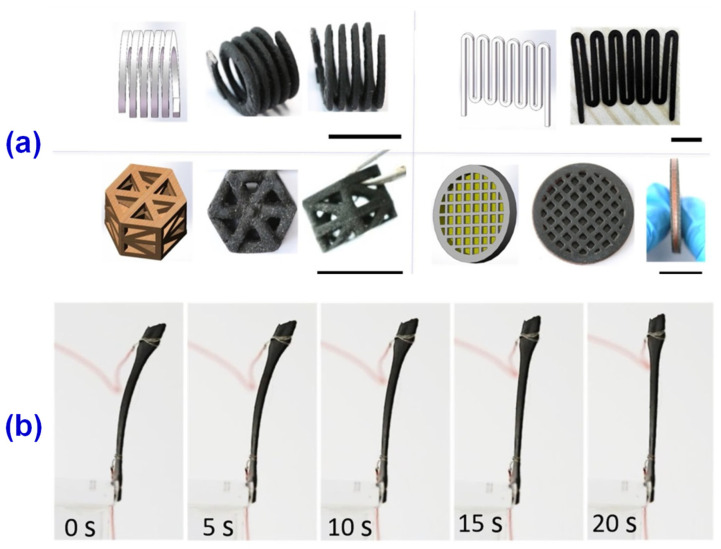
(**a**) Conductive structures of multi-walled carbon nanotube (MWCNT) nanocomposites and original resins. (**b**) Shape memory effect of the MWCNTs nanocomposites prepared by DLP 3D printing (ohmic heating process). Reprinted with permission from Ref. [101] Copyright 2017 Elsevier and Copyright Clearance Center.

The DLP 3D printing technology has been widely used to produce polymer products with high resolution and high productivity [98,99,100], where the application in electronics, batteries, and wearable devices has been heavily reported. Mu et al. [101] used commercially available acrylic-based UV curable resin and multi-walled carbon nanotubes (MWCNTs) as raw materials, dispersing multi-walled carbon nanotubes in DMF/Triton X-100 solution to explore the optimal ink ratio for conductivity and printing quality. They found that under the premise of comprehensive consideration of conductivity and printability, the optimal load of MWCNT was 0.3 wt% when the printing process parameters were adjusted to 19.05 μm and the illumination time was 40 s. At 0.3 wt% load of MWCNTs, the conductivity of the DLP 3D printed composite parts reaches up to 0.027 S/m. In addition, using DLP 3D printing technology, the conductive composite structures could be further prepared, such as hollow capacitance sensors, electrically activated shape memory composites, and stretchable circuits, as illustrated in Figure 12, which shows the versatility of DLP 3D printing for conductive composite structures. These demonstrations show that the DLP 3D printing technology can enable the material to be applied in many fields, such as electronics, wearable devices, soft robots, and so on.

**Figure 13:**
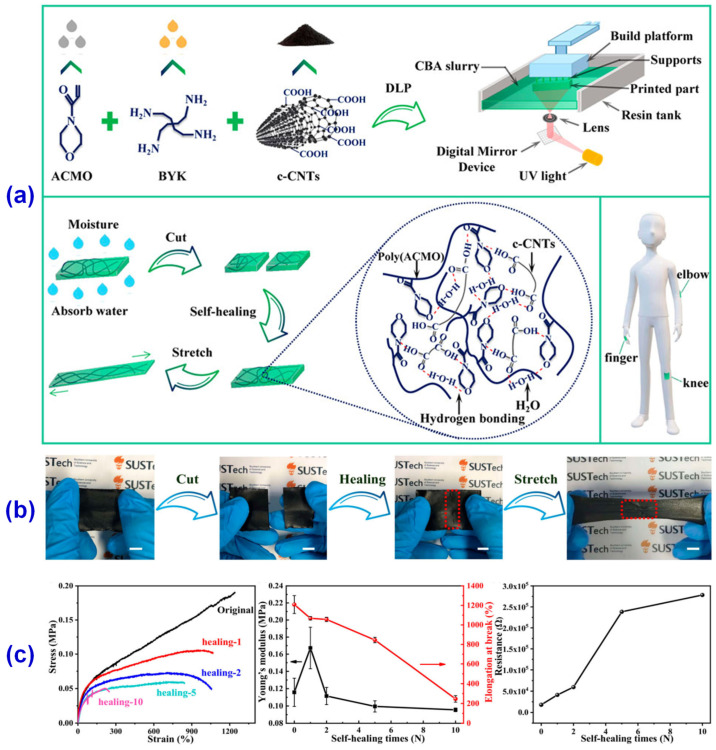
(**a**) Preparation, printing process, and application of CBA nano-suspension. (**b**) Stretching and self-healing process of CBA film. (**c**) (left) Stress-strain curves of the original, first, second, fifth, and tenth self-healing, (middle) Young’s modulus and elongation at break curves, and (right) resistance self-healing time curve. Reprinted from Ref. [105].

There are growing requirements for the multi-function of wearable electronic devices in the contemporary market, which will be a huge challenge for the development of the core strain sensor of electronic devices [102,103]. The traditional strain sensor manufacturing process takes too long, so it is difficult for large-scale industrial production, and it is difficult to wear in parts such as joints due to insufficient retraction performance. In the process of use, it is easy to cause the failure of the whole strain sensor due to a slight failure [104]. Based on these problems, Guo et al. [105] proposed a 3D printing strain sensor with good scalability and self-healing ability. They used carboxyl carbon nanotubes (c-CNTs) with better dispersion properties as a conductive medium and N-acryloylmorpholine (ACMO) with low viscosity, low volatility, and excellent water solubility to form hydrogen bonds among water, carbon group, and amino group, which would enhance the tensile properties and inherent self-healing ability of the composites. Moreover, the c-CNTs/BYK/ACMO (CBA) suspension was further prepared by a simple water absorption process, and a new strain sensor was produced in the DLP 3D printer, as shown in Figure 13. The new strain sensor shows good stretching ability and internal self-healing ability and can provide stable signal information, which would have great potential applications in the fields of stretchable devices, electronic skin, wearable electronic devices, and so on.

### 3.4. Other Printing Technologies

**Figure 14:**
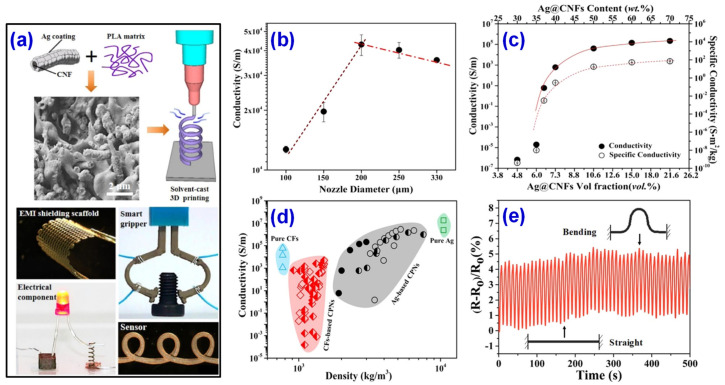
(**a**) Preparation of Ag@CNFs/polylactic acid (PLA) composites, SEM and 3D printing application of the as-prepared Ag@CNFs/PLA composites. (**b**) Conductively versus nozzle size. (**c**) Conductivity and specific conductivity as a function of Ag@CNF loading. (**d**) Property space map of conductivity with respect to density that compares the as-prepared (solid symbols) to other reported 3D printable conductive polymer nanocomposites (CPNs). (**e**) Plot of (R-R0)/R0 over time during the bending cyclic test of a filament extruded from a nozzle of 200 μm using Ag@CNF CPNs with 10.6 vol% fillers. Reprinted with permission from Ref. [114] Copyright 2019 American Chemical Society.

After years of development and later innovation, the 3D printing technology has derived new printing methods, such as biological 3D printing [106], and solvent casting 3D printing [107,108], etc. These new printing methods have been gradually applied in biological applications [109,110], electronics [111], wearable devices [112,113], and other fields. Conductive polymer nanocomposites (CPNs) prepared with traditional silver nanoparticles as fillers have good electrical conductivity, but the use of plenty of precious metal silver also restricts its application in industry. To solve this problem, Wei et al. [114] innovatively introduced hybrid nanofibers, namely Ag coated CNFs (Ag@CNFs) as a conductive filler, and designed 3D printable, lightweight, and highly conductive nanocomposites. The silver was deposited on the carbon nanotubes by chemical deposition to produce Ag@CNFs. The core-shell structure obtained combines the high aspect ratio of CNFs with the high conductivity and low contact resistance of Ag. The process has no by-product and high production efficiency. The product can be used immediately after production without any purification. These advantages indicate that the Ag@CNFs is a promising nano-filler for the construction of 3D printable CPN with highly adjustable electrical properties. In addition, Wei et al. [114] successfully demonstrated that using solvent casting 3D printing technology, the Ag@CNF/PLA conductive polymer nanocomposites can be directly used in a variety of demanding applications, including electronic components, strain sensors, and EMI shielding brackets. The preparation process, 3D printed products of the composite materials, and the corresponding properties are respectively presented in Figure 14. The composite materials are expected to be used in “on-demand” to design and build a series of electronic devices with superior performance, that are lightweight, and have shape memory, which can be used in more advanced applications from electronics to aerospace.

**Figure 15:**
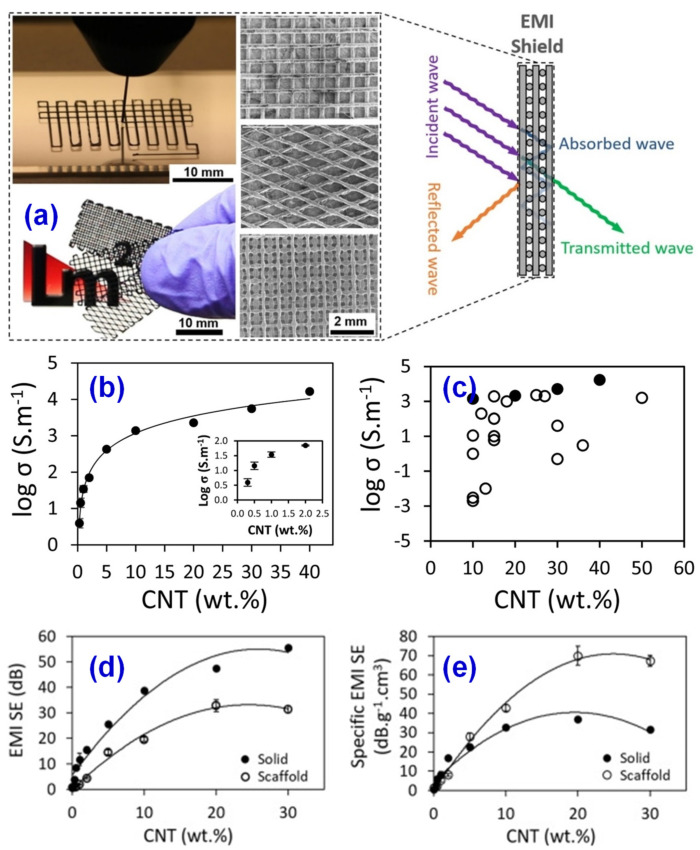
(**a**) 3D printed supports by using 200 μm inner diameter nozzle and their SEM. (**b**) Conductivity of CNT/PLA versus CNT load. (**c**) Conductivity of CNT/PLA comparing with the existed reports in the literatures. (**d**) Electromagnetic interference shielding effectiveness (EMI SE) and (**e**) specific EMI SE of average electromagnetic shielding performance of solid and support structures as a function of CNT load. Reprinted with permission from Ref. [108] Copyright 2017 Elsevier and Copyright Clearance Center.

Electronic equipment will continue to emit electromagnetic waves in the process of use, which could affect normal electromagnetic wave communication and even threaten people’s health [115,116]. Traditional electromagnetic shielding equipment is usually made of metal materials. Metal materials have a good shielding effect, but corrode easily and are difficult to regenerate, which limits the use of metal in electromagnetic shielding. Chizari et al. [117] used carbon nanotubes/polylactic acid (CNT/PLA) nanocomposites to prepare high conductivity 3D printable ink with conductivity up to 5000 S/m. Using the solvent casting 3D printing technology, the conductive grid structures were prepared, and then the effects of various parameters on the transparency and EMI shielding effectiveness of the products were further studied, as resulted in Figure 15. The results show that the EMI shielding effectiveness (EMI SE) value of CNT/PLA nanocomposites 3D printed in the form of three-dimensional scaffolds is significantly higher than that of CNT/PLA nanocomposites 3D printed in the form of hot-pressed solid. By changing the printing pattern and fiber spacing, the transparency of the scaffold can vary from ~0% to ~75%. This is also the first systematic study on EMI shielding using 3D printing technology. These results are very useful for the fabrication and structural optimization of EMI shielding with optical or transparent structures, such as in aerospace systems, portable electronic devices, or smart fabrics.

**Figure 16:**
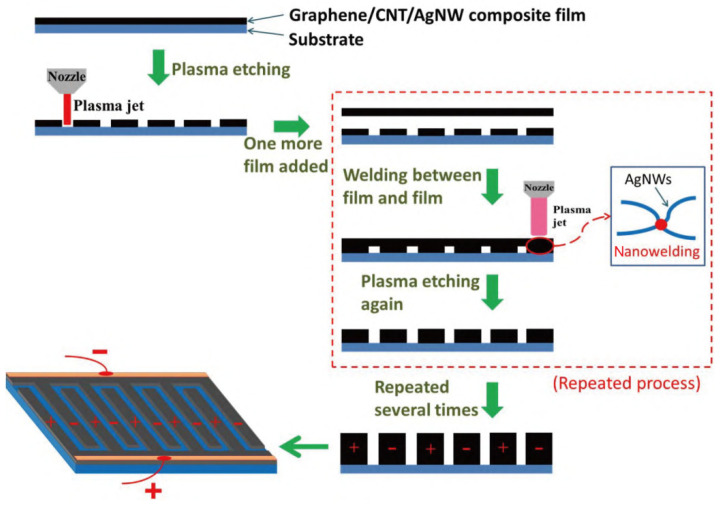
Schematic diagram of the fabrication processes of 3D printed GE/CNT/AgNW micro supercapacitors (MSCs). Reprinted with permission from Ref. [120] Copyright 2021 Springer Nature and Copyright Clearance Center.

**Figure 17:**
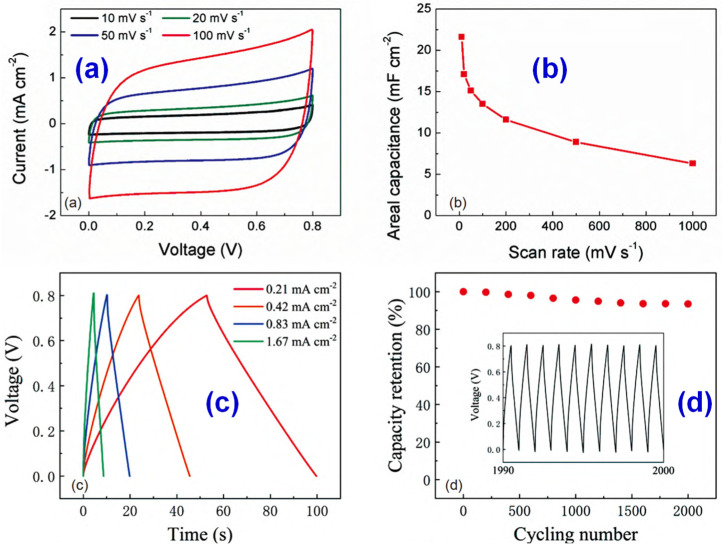
(**a**) CV curves of a 3D printed GE/CNT/AgNW MSC at scan rates of 10, 20, 50, and 100 V/s. (**b**) Areal capacitance as a function of scan rate. (**c**) GCD curves at different current densities. (**d**) Percent of retained capacitance as a function of the cycle number. Reprinted with permission from Ref. [120] Copyright 2021 Springer Nature and Copyright Clearance Center.

With the demand for portable electronic devices, the demand for micro supercapacitor (MSC) is greatly improved [118,119]. The energy density of the common thin-film MSC is low, which makes the endurance of the battery very poor. Increasing the thickness of the electrode is an effective way to improve the energy density. However, the research on the preparation methods of thick electrode MSCs is still very weak and the manufacturing of ideal MSCs still remains a challenge. Lang et al. [120] used a 3D printing method based on plasma jet to prepare high-performance MSCs with graphene (GE)/carbon nanotube (CNT)/silver nanowire (AgNW) electrode. The preparation process is shown in Figure 16. A certain amount of phosphorus was added in the preparation process to improve the electrochemical performance of graphene [121,122]. The capacitance of GE/CNT/AgNW MSC prepared by 3D printing was 21.3 mF/cm at the scanning rate of 0.01 V/s, showing good energy density. After 2000 cycles, 93.1% of the initial capacitance could still be retained. Figure 17 shows the test results, which demonstrate that the battery life is markedly improved and the service life is increased, indicating that using 3D printing technology to produce high-performance MSCs is a feasible and efficient preparation method, and has great development potential.

## 4. Conclusions and Outlook

The technology of additive manufacturing, 3D printing technology, has become the most popular advanced technology in the world in recent years. After more than 30 years of development, the technology of material addition manufacturing has been moving from laboratory to mass use and has been able to play a leading role in aerospace, modelling, fashion, and other fields. At present, there are many reports on 3D printable conductive carbon materials. Compared with metal conductive materials, conductive carbon materials have great advantages in price and lightweight quality, which means that the energy lost due to quality can be greatly reduced, which is of great significance for energy conservation and emission reduction. Based on the 3D printing technology, we can produce products of any structure in principle. The combination of conductive carbon material and 3D printing technology can produce a personalized motion sensor, which has great potential in the detection of human health. It can be integrated into textiles, as wearable devices, and then wearable devices can be used to observe the health of patients in real-time, which can greatly reduce the working intensity of medical staff. Compared with traditional metal shielding, it has obvious advantages in lightweight and structural diversification, which is of great significance to the stable transmission of communication signals, military defense, human health, and so on. It can design a variety of electronic components, to a large extent, save the cost of producing a small number of personalized accessories, and so on.

However, it is undeniable that 3D printing technology is not yet mature. Although it has great development potential, there are bottlenecks in the development of this technology, and its the main disadvantages are as follows:

(1) **The price of raw materials is too expensive.** Taking plastics as an example, the price of traditional injection molding plastics is 2–3 dollars/kg, while the price of 3D printing plastics is 175–250 dollars/kg. The high cost of raw materials is ultimately reflected in the high price of products, which is difficult to accept in the current market.
(2) **There are few printable materials, which makes it difficult to meet demand.** There are only 20 kinds of monomer material for 3D printing, and only 140 kinds of material can be selected after mixing, which is too limited compared with thousands of materials in various application fields.
(3) **The existing materials are insufficiently optimized.** Additive manufacturing has higher requirements of the performance and applicability of materials, the properties of materials before and after printing remain stable, and can meet the requirements of continuous production, which requires further optimization of materials.
(4) **Lack of color options.** The color options for 3D printing are very limited. In addition, the color of composite materials with conductive carbon materials is always black, which restricts the use of 3D printing technology in creative industries.
(5) **Deficiency of standard/certification.** At present, there is no standardized database of material performance, which means it is not possible to certify the level of equipment or new materials.

From the development bottleneck of 3D printing technology, we can see that the future development direction of 3D printable conductive carbon materials will further reduce the production cost of raw materials, find and use cheaper matrix materials with excellent performance, and improve the competitiveness of products. To meet the requirements of all walks of life for conductive materials, we need to develop a variety of new 3D printable conductive carbon materials to optimize the performance of materials. It is also necessary to further establish and improve the standards and database of 3D printable conductive carbon materials, which has a measurable standard for the development of new conductive materials. At the same time, this can quickly select suitable materials for production, saving time spent in finding suitable materials. These problems need to be solved urgently in the promotion and application of 3D printable conductive carbon materials in the future, and need to be studied by researchers in the future.

## Data Availability

The study did not report any data.

## References

[1] Kodama H. (1981). Automatic method for fabricating a 3-dimensional plastic model with photo-hardening polymer. Rev. Sci. Instrum..

[2] Freedman D.H. (2012). Layer by layer. Technol. Rev..

[3] Gawel T.G. (2020). Review of additive manufacturing methods. Solid State Phenom..

[4] Pearce J.M. (2012). Building research equipment with free, open-source hardware. Science.

[5] Bogue R. (2013). 3D printing: The dawn of a new era in manufacturing?. Assem. Autom..

[6] Campbell T.A., Ivanova O.S. (2013). 3D printing of multifunctional nanocomposites. Nano Today.

[7] Christ J.F., Aliheidari N., Ameli A., Poetschke P. (2017). 3D printed highly elastic strain sensors of multiwalled carbon nanotube/thermoplastic polyurethane nanocomposites. Mater. Des..

[8] Mahmoodi M., Arjmand M., Sundararaj U., Park S. (2011). The electrical conductivity and electromagnetic interference shielding of injection molded multi-walled carbon nanotube/polystyrene composites. Carbon.

[9] Palenzuela C.L.M., Novotny F., Krupicka P., Sofer Z., Pumera M. (2018). 3D-printed graphene/polylactic acid electrodes promise high sensitivity in electroanalysis. Anal. Chem..

[10] Ahn B.Y., Duoss E.B., Motala M.J., Guo X., Park S.I., Xiong Y., Yoon J., Nuzzo R.G., Rogers J.A., Lewis J.A. (2009). Omnidirectional printing of flexible, stretchable, and spanning silver microelectrodes. Science.

[11] Rymansaib Z., Iravani P., Emslie E., Medvidovic-Kosanovic M., Sak-Bosnar M., Verdejo R., Marken F. (2016). All-polystyrene 3D-printed electrochemical device with embedded carbon nanofiber-graphite-polystyrene composite conductor. Electroanalysis.

[12] Muth J.T., Vogt D.M., Truby R.L., Menguc Y., Kolesky D.B., Wood R.J., Lewis J.A. (2014). Embedded 3D printing of strain sensors within highly stretchable elastomers. Adv. Mater..

[13] Mu Q.Y., Dunn C.K., Wang L., Dunn M.L., Qi H.J., Wang T.J. (2017). Thermal cure effects on electromechanical properties of conductive wires by direct ink write for 4D printing and soft machines. Smart Mater. Struct..

[14] Leigh S.J., Bradley R.J., Purssell C.P., Billson D.R., Hutchins D.A. (2012). A simple, low-cost conductive composite material for 3D printing of electronic sensors. PLoS ONE.

[15] Zhang J., Yang B., Fu F., You F., Dong X., Dai M. (2017). Resistivity and its anisotropy characterization of 3D-printed acrylonitrile butadiene styrene copolymer (ABS)/carbon black (CB) composites. Appl. Sci..

[16] Balandin A.A. (2011). Thermal properties of graphene and nanostructured carbon materials. Nat. Mater..

[17] Zhang Q.Q., Xu Y., Yang Y., Li L.S., Song C.R., Su X. (2018). Conductive mechanism of carbon black/polyimide composite films. J. Polym. Eng..

[18] Al-Saleh M.H., Sundararaj U. (2009). Electromagnetic interference shielding mechanisms of CNT/polymer composites. Carbon.

[19] Cao M.S., Wang X.X., Cao W.Q., Yuan J. (2015). Ultrathin graphene: Electrical properties and highly efficient electromagnetic interference shielding. J. Mater. Chem. C.

[20] Ornaghi H.L., Neves R.M., Monticeli F.M., Almeida J.H.S. (2020). Viscoelastic characteristics of carbon fiber-reinforced epoxy filament wound laminates. Compos. Commun..

[21] Khodabakhshi S., Fulvio P.F., Andreoli E. (2020). Carbon black reborn: Structure and chemistry for renewable energy harnessing. Carbon.

[22] Donnet J.B., Bansal R.C., Wang M.J. (1993). Carbon Black: Science and Technology.

[23] Cheah K., Forsyth M., Simon G.P. (2000). Processing and morphological development of carbon black filled conducting blends using a binary host of poly(styrene co-acrylonitrile) and poly(styrene). J. Polym. Sci. Part B Polym. Phys..

[24] Gao C., Zhang S.L., Lin Y.J., Li F., Guan S.W., Jiang Z.H. (2015). High-performance conductive materials based on the selective location of carbon black in poly(ether ether ketone)/polyimide matrix. Compos. Part B Eng..

[25] Chen J.W., Cui X.H., Sui K.Y., Zhu Y.T., Jiang W. (2017). Balance the electrical properties and mechanical properties of carbon black filled immiscible polymer blends with a double percolation structure. Compos. Sci. Technol..

[26] Das T.K., Ghosh P., Das N.C. (2019). Preparation, development, outcomes, and application versatility of carbon fiber-based polymer composites: A review. Adv. Compos. Hybrid Mater..

[27] Hirota K., Fueki K., Shindo K., Nakai Y. (1959). Studies on the state of formic acid adsorbed on silica and alumina by a combined method of nuclear magnetic resonance and infrared absorption. Bull. Chem. Soc. Jpn..

[28] Ameli A., Jung P.U., Park C.B. (2013). Electrical properties and electromagnetic interference shielding effectiveness of polypropylene/carbon fiber composite foams. Carbon.

[29] Iijima S. (1991). Helical microtubules of graphitic carbon. Nature.

[30] Park J.G., Smithyman J., Lin C.Y., Cooke A., Kismarahardja A.W., Li S., Liang R., Brooks J.S., Zhang C., Wang B. (2009). Effects of surfactants and alignment on the physical properties of single-walled carbon nanotube buckypaper. J. Appl. Phys..

[31] Yu M.F., Lourie O., Dyer M.J., Moloni K., Kelly T.F., Ruoff R.S. (2000). Strength and breaking mechanism of multiwalled carbon nanotubes under tensile load. Science.

[32] Li S., Park J.G., Liang Z.Y., Siegrist T., Liu T., Zhang M., Cheng Q.F., Wang B., Zhang C. (2012). In situ characterization of structural changes and the fraction of aligned carbon nanotube networks produced by stretching. Carbon.

[33] Thostenson E.T., Li C.Y., Chou T.W. (2005). Nanocomposites in context. Compos. Sci. Technol..

[34] Zhang W.Y., Hou L.Y., Zhou H.H. (2020). Electrochemically treated graphite/poly(3,4-ethylenedioxythiophene)-carbon nanotubes electrode: Facile preparation and remarkable enhancement in electrochemical performances. Fuller. Nanotubes Carbon Nanostruct..

[35] Tian F., Zhong S.W., Nie W., Zeng M., Chen B.M., Liu X.L. (2020). Multi-walled carbon nanotubes prepared with low-cost Fe-Al bimetallic catalysts for high-rate rechargeable Li-ion batteries. J. Solid State Electrochem..

[36] Gan D.F., Dou J.B., Huang Q., Huang H.Y., Chen J.Y., Liu M.Y., Qi H.X., Yang Z.Y., Zhang X.Y., Wei Y. (2020). Carbon nanotubes-based polymer nanocomposites: Bio-mimic preparation and methylene blue adsorption. J. Environ. Chem. Eng..

[37] Faba L., Garcés D., Díaz E., Ordóñez S. (2019). Carbon materials as phase-transfer promoters for obtaining 5-hydroxymethylfurfural from cellulose in a biphasic system. ChemSusChem.

[38] Dey B., Ahmad M.W., Almezeni A., Sarkhel G., Bag D.S., Choudhury A. (2020). Enhancing electrical, mechanical, and thermal properties of polybenzimidazole by 3D carbon nanotube@graphene oxide hybrid. Compos. Commun..

[39] Shimizu T., Kishi R., Kobashi K., Morimoto T., Okazaki T., Yamada T., Hata K. (2020). Improved thermal stability of silicone rubber nanocomposites with low filler content, achieved by well-dispersed carbon nanotubes. Compos. Commun..

[40] Chen C., Liu J.W., Li X.W., Wen Y.F., Li X.J., Shi D.A., Xue Z.G., Xie X.L. (2019). Epoxy/ionic liquid-like mwcnts composites with improved processability and mechanical properties. Compos. Commun..

[41] Wang G.X., Yu Q.Z., Hu Y.M., Zhao G.Y., Chen J.W., Li H., Jiang N., Hu D.W., Xu Y.Q., Zhu Y.T. (2020). Influence of the filler dimensionality on the electrical, mechanical and electromagnetic shielding properties of isoprene rubber-based flexible conductive composites. Compos. Commun..

[42] Brownson D.A.C., Kelly P.J., Banks C.E. (2015). In situ electrochemical characterisation of graphene and various carbon-based electrode materials: An internal standard approach. RSC Adv..

[43] Li W., Tan C., Lowe M.A., Abruna H.D., Ralph D.C. (2011). Electrochemistry of individual monolayer graphene sheets. ACS Nano.

[44] Novoselov K.S., Geim A.K., Morozov S.V., Jiang D., Zhang Y., Dubonos S.V., Grigorieva I.V., Firsov A.A. (2004). Electric field effect in atomically thin carbon films. Science.

[45] Lee C., Wei X.D., Kysar J.W., Hone J. (2008). Measurement of the elastic properties and intrinsic strength of monolayer graphene. Science.

[46] Service R.F. (2009). Materials science carbon sheets an atom thick give rise to graphene dreams. Science.

[47] Novoselov K.S., Jiang Z., Zhang Y., Morozov S.V., Stormer H.L., Zeitler U., Maan J.C., Boebinger G.S., Kim P., Geim A.K. (2007). Room-temperature quantum hall effect in graphene. Science.

[48] Li X.H., Li X.F., Liao K.N., Min P., Liu T., Dasari A., Yu Z.Z. (2016). Thermally annealed anisotropic graphene aerogels and their electrically conductive epoxy composites with excellent electromagnetic interference shielding efficiencies. ACS Appl. Mater. Interfaces.

[49] Yang J.M., Liao X., Wang G., Chen J., Guo F.M., Tang W.Y., Wang W., Yan Z.H., Li G.X. (2020). Gradient structure design of lightweight and flexible silicone rubber nanocomposite foam for efficient electromagnetic interference shielding. Chem. Eng. J..

[50] Li J.T., Zhang G.C., Ma Z.L., Fan X.L., Fan X., Qin J.B., Shi X.T. (2016). Morphologies and electromagnetic interference shielding performances of microcellular epoxy/multi-wall carbon nanotube nanocomposite foams. Compos. Sci. Technol..

[51] Jiang Q.Y., Liao X., Yang J.M., Wang G., Chen J., Tian C.X., Li G.X. (2020). A two-step process for the preparation of thermoplastic polyurethane/graphene aerogel composite foams with multi-stage networks for electromagnetic shielding. Compos. Commun..

[52] Ngo T.D., Kashani A., Imbalzano G., Nguyen K.T.Q., Hui D. (2018). Additive manufacturing (3D printing): A review of materials, methods, applications and challenges. Compos. Part B Eng..

[53] Tian X.Y., Liu T.F., Yang C.C., Wang Q.R., Li D.C. (2016). Interface and performance of 3D printed continuous carbon fiber reinforced PLA composites. Compos. Part A Appl. Sci. Manuf..

[54] Ning F.D., Cong W.L., Qiu J.J., Wei J.H., Wang S.R. (2015). Additive manufacturing of carbon fiber reinforced thermoplastic composites using fused deposition modeling. Compos. Part B Eng..

[55] Seto Y., Sado A., Asami K., Hanada A., Umehara M., Akiyama K., Yamaguchi S. (2014). Carlactone is an endogenous biosynthetic precursor for strigolactones. Proc. Natl. Acad. Sci. USA.

[56] Rombouts M., Kruth J.P., Froyen L., Mercelis P. (2006). Fundamentals of selective laser melting of alloyed steel powders. CIRP Ann. Manuf. Technol..

[57] Ponnamma D., Yin Y., Salim N., Parameswaranpillai J., Thomas S., Hameed N. (2021). Recent progress and multifunctional applications of 3D printed graphene nanocomposites. Compos. Part B Eng..

[58] Xu W.P., Miao L.T., Liu L.G. (2017). Review on Structure Optimization in 3D Printing. J. Comput. Aided Des. Comput. Graph..

[59] Dou H., Cheng Y.Y., Ye W.G., Zhang D.H., Li J.J., Miao Z.J., Rudykh S. (2020). Effect of process parameters on tensile mechanical properties of 3D printing continuous carbon fiber-reinforced PLA composites. Materials.

[60] Zhang X., Wang J.L., Liu T.X. (2021). 3D printing of polycaprolactone-based composites with diversely tunable mechanical gradients via multi-material fused deposition modeling. Compos. Commun..

[61] Zhang X., Fan W., Liu T.X. (2020). Fused deposition modeling 3D printing of polyamide-based composites and its applications. Compos. Commun..

[62] Saharudin M.S., Hajnys J., Kozior T., Gogolewski D., Zmarzly P. (2021). Quality of surface texture and mechanical properties of PLA and PA-based material reinforced with carbon fibers manufactured by FDM and CFF 3D printing technologies. Polymers.

[63] Zhang X., Wang J.L. (2020). Controllable interfacial adhesion behaviors of polymer-on-polymer surfaces during fused deposition modeling 3D printing process. Chem. Phys. Lett..

[64] Peng X.S., Zhang M.M., Guo Z.C., Sang L., Hou W.B. (2020). Investigation of processing parameters on tensile performance for FDM-printed carbon fiber reinforced polyamide 6 composites. Compos. Commun..

[65] Wang X., Jiang M., Zhou Z.W., Gou J.H., Hui D. (2017). 3D printing of polymer matrix composites: A review and prospective. Compos. Part B Eng..

[66] Fostert C.W., Down M.P., Zhang Y., Ji X.B., Rowley-Neale S.J., Smith G.C., Kelly P.J., Banks C.E. (2017). 3D printed graphene based energy storage devices. Sci. Rep..

[67] Bárnik F., Vaško M., Handrik M., Dorčiak F., Majko J. (2019). Comparing mechanical properties of composites structures on Onyx base with different density and shape of fill. Transp. Res. Procedia.

[68] Strozzi M., Giacomobono R., Rubini R., Cocconcelli M. (2020). Preliminary orthotropic elastic model for the study of natural frequencies and mode shapes of a 3D printed Onyx thin circular cylindrical shell. Int. J. Mech. Control.

[69] Ghebretinsae F., Mikkelsen O., Akessa A.D. (2019). Strength analysis of 3D printed carbon fibre reinforced thermoplastic using experimental and numerical methods. IOP Conf. Ser. Mater. Sci. Eng..

[70] Huang P., Xia Z.D., Cui S. (2018). 3D printing of carbon fiber-filled conductive silicon rubber. Mater. Des..

[71] Daver F., Baez E., Shanks R.A., Brandt M. (2016). Conductive polyolefin-rubber nanocomposites with carbon nanotubes. Compos. Part A Appl. Sci. Manuf..

[72] Lukić M., Clarke J., Tuck C., Whittow W., Wells G. (2016). Printability of elastomer latex for additive manufacturing or 3D printing. J. Appl. Polym. Sci..

[73] Spahiu T., Fafenrot S., Grimmelsmann N., Piperi E., Shehi E., Ehrmann A. (2017). Varying fabric drape by 3D-imprinted patterns for garment design. IOP Conf. Ser. Mater. Sci. Eng..

[74] Shin D.G., Kim T.H., Kim D.E. (2017). Review of 4D printing materials and their properties. Int. J. Precis. Eng. Manuf. Green Technol..

[75] Sanatgar R.H., Cayla A., Campagne C., Nierstrasz V. (2019). Morphological and electrical characterization of conductive polylactic acid based nanocomposite before and after FDM 3D printing. J. Appl. Polym. Sci..

[76] Sanatgar R.H., Campagne C., Nierstrasz V. (2017). Investigation of the adhesion properties of direct 3D printing of polymers and nanocomposites on textiles: Effect of FDM printing process parameters. Appl. Surf. Sci..

[77] Ransley M., Smitham P., Miodownik M. (2017). Active chainmail fabrics for soft robotic applications. Smart Mater. Struct..

[78] Pei E.J., Shen J.S., Watling J. (2015). Direct 3D printing of polymers onto textiles: Experimental studies and applications. Rapid Prototyp. J..

[79] Mpofu N.S., Mwasiagi J.I., Nkiwane L.C., Njuguna D. (2019). Use of regression to study the effect of fabric parameters on the adhesion of 3D printed PLA polymer onto woven fabrics. Fash. Text..

[80] Grimmelsmann N., Kreuziger M., Korger M., Meissner H., Ehrmann A. (2018). Adhesion of 3D printed material on textile substrates. Rapid Prototyp. J..

[81] Eutionnat-Diffo P.A., Chen Y., Guan J.P., Cayla A., Campagne C., Zeng X.Y., Nierstrasz V. (2019). Stress, strain and deformation of poly-lactic acid filament deposited onto polyethylene terephthalate woven fabric through 3D printing process. Sci. Rep..

[82] Eutionnat-Diffo P.A., Chen Y., Guan J.P., Cayla A., Campagne C., Zeng X.Y., Nierstrasz V. (2020). Optimization of adhesion of poly lactic acid 3D printed onto polyethylene terephthalate woven fabrics through modelling using textile properties. Rapid Prototyp. J..

[83] Eutionnat-Diffo P.A., Cayla A., Chen Y., Guan J.P., Nierstrasz V., Campagne C. (2020). Development of flexible and conductive immiscible thermoplastic/elastomer monofilament for smart textiles applications using 3D printing. Polymers.

[84] Evans R.S., Bourell D.L., Beaman J.J., Campbell M.I. (2005). Rapid manufacturing of silicon carbide composites. Rapid Prototyp. J..

[85] Goodridge R.D., Tuck C.J., Hague R.J.M. (2011). Laser sintering of polyamides and other polymers. Prog. Mater. Sci..

[86] Olakanmi E.O., Cochrane R.F., Dalgarno K.W. (2015). A review on selective laser sintering/melting (SLS/SLM) of aluminium alloy powders: Processing, microstructure, and properties. Prog. Mater Sci..

[87] Bose S., Bhattacharyya A.R., Kulkarni A.R., Poetschke P. (2009). Electrical, rheological and morphological studies in co-continuous blends of polyamide 6 and acrylonitrile-butadiene-styrene with multiwall carbon nanotubes prepared by melt blending. Compos. Sci. Technol..

[88] Araby S., Meng Q.S., Zhang L.Q., Kang H.L., Majewski P., Tang Y.H., Ma J. (2014). Electrically and thermally conductive elastomer/graphene nanocomposites by solution mixing. Polymer.

[89] Zhan Y.H., Lavorgna M., Buonocore G., Xia H.S. (2012). Enhancing electrical conductivity of rubber composites by constructing interconnected network of self-assembled graphene with latex mixing. J. Mater. Chem..

[90] Lao S.C., Koo J.H., Moon T.J., Londa M., Ibeh C.C., Wissler G.E., Pilato L.A. (2011). Flame-retardant polyamide 11 nanocomposites: Further thermal and flammability studies. J. Fire Sci..

[91] Jacobs C.J., Tate J.S., Olson B., Theodoropoulou N., Koo J.H. (2012). Thermal characterization of polyamide 11/nanographene platelet nanocomposites. J. Nanosci. Nanotechnol..

[92] Athreya S.R., Kalaitzidou K., Das S. (2010). Processing and characterization of a carbon black-filled electrically conductive nylon-12 nanocomposite produced by selective laser sintering. Mater. Sci. Eng. A.

[93] Li Z.C., Wang Z.H., Gan X.P., Fu D.H., Fei G.X., Xia H.S. (2017). Selective laser sintering 3D printing: A way to construct 3D electrically conductive segregated network in polymer matrix. Macromol. Mater. Eng..

[94] Florence J.M., Yoder L.A. (1996). Display system architectures for digital micromirror device (DMD(TM)) based projectors. Proc. Soc. Photo-Opt. Instrum. Eng. (SPIE).

[95] Quan H.Y., Zhang T., Xu H., Luo S., Nie J., Zhu X.Q. (2020). Photo-curing 3D printing technique and its challenges. Bioact. Mater..

[96] Ligon S.C., Liska R., Stampfl J., Gurr M., Mulhaupt R. (2017). Polymers for 3D printing and customized additive manufacturing. Chem. Rev..

[97] Peng S.Q., Wang Z., Lin J.B., Miao J.T., Zheng L.H., Yang Z., Weng Z.X., Wu L.X. (2020). Tailored and highly stretchable sensor prepared by crosslinking an enhanced 3D printed UV-curable sacrificial mold. Adv. Funct. Mater..

[98] Li S.Y., Yuan S.Q., Zhu J.H., Wang C., Li J., Zhang W.H. (2020). Additive manufacturing-driven design optimization: Building direction and structural topology. Addit. Manuf..

[99] Sun J.X., Binner J., Bai J.M. (2020). 3D printing of zirconia via digital light processing: Optimization of slurry and debinding process. J. Eur. Ceram. Soc..

[100] Tan L.J.Y., Zhu W., Zhou K. (2020). Recent progress on polymer materials for additive manufacturing. Adv. Funct. Mater..

[101] Mu Q.Y., Wang L., Dunn C.K., Kuang X., Duan F., Zhang Z., Qi H.J., Wang T.J. (2017). Digital light processing 3D printing of conductive complex structures. Addit. Manuf..

[102] Wang R.J., Tan Z.Q., Zhong W.B., Liu K., Li M.F., Chen Y.L., Wang W.W., Wang D. (2020). Polypyrrole (PPy) attached on porous conductive sponge derived from carbonized graphene oxide coated polyurethane (PU) and its application in pressure sensor. Compos. Commun..

[103] Liang G.J., Hu H.B., Liao L., He Y.B., Ye C.H. (2017). Highly flexible and bright electroluminescent devices based on Ag nanowire electrodes and top-emission structure. Adv. Electron. Mater..

[104] Jing X., Mi H.Y., Lin Y.J., Enriquez E., Peng X.F., Turng L.S. (2018). Highly stretchable and biocompatible strain sensors based on mussel-inspired super-adhesive self-healing hydrogels for human motion monitoring. ACS Appl. Mater. Interfaces.

[105] Guo B.B., Ji X.Z., Chen X.T., Li G., Lu Y.G., Bai J.M. (2020). A highly stretchable and intrinsically self-healing strain sensor produced by 3D printing. Virtual Phys. Prototyp..

[106] Yuan H., Xing K., Hsu H.Y. (2018). Trinity of three-dimensional (3D) scaffold, vibration, and 3D printing on cell culture application: A systematic review and indicating future direction. Bioengineering.

[107] Kloft H., Krauss H.W., Hack N., Herrmann E., Neudecker S., Varady P.A., Lowke D. (2020). Influence of process parameters on the interlayer bond strength of concrete elements additive manufactured by shotcrete 3D printing (SC3DP). Cem. Concr. Res..

[108] Chizari K., Arjmand M., Liu Z., Sundararaj U., Therriault D. (2017). Three-dimensional printing of highly conductive polymer nanocomposites for EMI shielding applications. Mater. Today Commun..

[109] Gong Y., Wang F., Al-Furjan M.S.H., Shan L.J., He J.Y., Bian X.J., Bi Z.K., Liu H.A., Li W.X., Shao H.F. (2020). Experimental investigation and optimal 3D bioprinting parameters of SA-Gel porous cartilage scaffold. Appl. Sci..

[110] Cuellar J.S., Plettenburg D., Zadpoor A.A., Breedveld P., Smit G. (2021). Design of a 3D-printed hand prosthesis featuring articulated bio-inspired fingers. Proc. Inst. Mech. Eng. Part H.

[111] Jakus A.E., Shah R.N. (2017). Multi and mixed 3D-printing of graphene-hydroxyapatite hybrid materials for complex tissue engineering. J. Biomed. Mater. Res. Part A.

[112] Marsalek P., Sotola M., Rybansky D., Repa V., Halama R., Fusek M., Prokop J. (2020). Modeling and testing of flexible structures with selected planar patterns used in biomedical applications. Materials.

[113] Abdollahi S., Markvicka E.J., Majidi C., Feinberg A.W. (2020). 3D printing silicone elastomer for patient-specific wearable pulse oximeter. Adv. Healthc. Mater..

[114] Wei H.Q., Cauchy X., Navas I.O., Abderrafai Y., Chizari K., Sundararaj U., Liu Y.J., Leng J., Therriault D. (2019). Direct 3D printing of hybrid nanofiber-based nanocomposites for highly conductive and shape memory applications. ACS Appl. Mater. Interfaces.

[115] Lawrentschuk N., Bolton D.M. (2004). Experience and attitudes of final-year medical students to digital rectal examination. Med. J. Aust..

[116] Wang H.B., Li N., Wang W., Shi J., Xu Z.W., Liu L.S., Hu Y.L., Jing M.L., Liu L.Y., Zhang X.X. (2019). Bead nano-necklace spheres on 3D carbon nanotube scaffolds for high-performance electromagnetic-interference shielding. Chem. Eng. J..

[117] Maity S., Chatterjee A. (2018). Conductive polymer-based electro-conductive textile composites for electromagnetic interference shielding: A review. J. Ind. Text..

[118] Zeng W., Shu L., Li Q., Chen S., Wang F., Tao X.M. (2014). Fiber-based wearable electronics: A review of materials, fabrication, devices, and applications. Adv. Mater..

[119] Yao Y.F., Dong H.L., Hu W.P. (2016). Charge Transport in Organic and Polymeric Semiconductors for Flexible and Stretchable Devices. Adv. Mater..

[120] Liu L., Lu J.Y., Long X.L., Zhou R., Liu Y.Q., Wu Y.T., Yan K.W. (2021). 3D printing of high-performance micro-supercapacitors with patterned exfoliated graphene/carbon nanotube/silver nanowire electrodes. Sci. China Technol. Sci..

[121] Zhang C.Z., Mahmood N., Yin H., Liu F., Hou Y.L. (2013). Synthesis of phosphorus-doped graphene and its multifunctional applications for oxygen reduction reaction and lithium ion batteries. Adv. Mater..

[122] Wen Y.Y., Wang B., Huang C.C., Wang L.Z., Hulicova-Jurcakova D. (2015). Synthesis of phosphorus-doped graphene and its wide potential window in aqueous supercapacitors. Chem. Eur. J..

